# Electrophysical behavior of ion-conductive organic-inorganic polymer system based on aliphatic epoxy resin and salt of lithium perchlorate

**DOI:** 10.1186/1556-276X-9-674

**Published:** 2014-12-12

**Authors:** Liubov Matkovska, Maksym Iurzhenko, Yevgen Mamunya, Olga Matkovska, Valeriy Demchenko, Eugene Lebedev, Gisele Boiteux, Anatoli Serghei

**Affiliations:** Institute of Macromolecular Chemistry of National Academy of Sciences of Ukraine, Kyiv, 02160 Ukraine; IMP@LYON1, Ingénierie des Matériaux Polymères, UMR CNRS 5223, Université Lyon 1, Université de Lyon, Lyon, 69007 France

**Keywords:** Ion-conductive polymers, Epoxy resin, Lithium perchlorate, Glass transition temperature, Electrical and dielectric characteristics

## Abstract

**Abstract:**

In the present work, ion-conductive hybrid organic-inorganic polymers based on epoxy oligomer of diglycide aliphatic ester of polyethylene glycol (DEG) and lithium perchlorate (LiClO_4_) were synthesized. The effect of LiClO_4_ content on the electrophysical properties of epoxy polymers has been studied by differential scanning calorimetry (DSC) and broadband dielectric spectroscopy (BDS). The effect of LiClO_4_ content on the structure has been studied by wide-angle X-ray scattering (WAXS). It was found that LiClO_4_ impacts on the structure of the synthesized hybrid epoxy polymers, probably, by formation of coordinative complexes {ether oxygen-lithium cations-ether oxygen} as evidenced from a significant increase in their glass transition temperatures with increasing LiClO_4_ concentration and WAXS studies. The presence of ether oxygen in DEG macromolecules provides a transfer mechanism of the lithium cations with the ether oxygen similar to polyethylene oxide (PEO). Thus, the obtained hybrid polymers have high values of ionic conductivity *σ'* (approximately 10^−3^ S/cm) and permittivity *ϵ'* (6 × 10^5^) at elevated temperatures (200°С). On the other hand, DEG has higher heat resistance compared to PEO that makes these systems perspective as solid polymer electrolytes able to operate at high temperature.

**PACS:**

81.07.Pr; 62.23.St; 66.30.hk

## Background

Nowadays, one of the most important research directions in the development and creation of functional polymeric materials is the search of new solid electroactive polymers with high ionic conductivity at elevated temperatures. Particularly, the widening range of materials, which can be used for this purpose, is relevant [1]. For example, composite materials based on polymers and inorganic components are used for ion-conductive membranes production and further application in batteries, electrolyzers and fuel cells [2–8]. Usually, they have their advantages as well as disadvantages [1, 5] and only partially satisfy needs of industry in ion-conductive materials.

A considerable attention to the creation of new proton-conductive materials for fuel cell membranes is paid. For example, in [9] a membrane on the basis of the functionalized polyvinyl fluoride with phosphotungstic acid (PWA) additive that acts as a source of protons was proposed, and due to adding certain modifiers, the conductivity σ ≈ 10^−3^ to 10^−2^ S/cm at elevated temperatures of 60°C to 80°C was achieved. In [10] proton-conducting membrane based on sulfonated poly(phthalazinone ether ketone) (SPFEK) that holds PWA was produced. In this case the conductivity σ ≈ 0.17 S/cm at 80°C that is higher than that of Nafion 117 was achieved. The sulfurization of PFEK promoted fixing of PWA atoms in the polymer structure and prevented their leaching from the wet sample. Similar results were obtained for the sulfurized poly(ether ether ketone) (SPEEK) with PWA, wherein the proton conductivity was higher than 10^−3^ S/cm at 95% of the relative humidity and in the temperature range of 50°C to 100°C [11]. Proton-conductive membrane for methanol fuel cell based on chitosan and PWA in a fully hydrated state possessed conductivity of 10^−2^ S/cm at temperatures of 20°C to 80°C [12]. The authors of [13] synthesized the polymeric material based on the polybenzimidazole and phosphoric acid with proton conductivity σ ≈ 6 × 10^−2^ S/cm with the relative moisture content of 5% to 10% in the temperature range of 160°C to 200°C that is comparable to Nafion 117 at 80% of moisture content in the temperature range of 25°C to 80°C. The high ionic conductivity can be also achieved by dispersing various salts in a polymer matrix at molecular level, wherein conductivity is provided by the mobility of the metallic ions [14–18]. In general, the lithium salts are often used for creating the ion-conducting systems due to the smallest size of lithium ions, low-potential energy, and, correspondingly, low activation energy of the charge transfer [5, 19–21].

Thus, the study of the structure, mechanisms of ionic conductivity, and permittivity in such composite systems is highly important for the development of new effective electroactive materials. It is known that the use of organic compounds such as olіgoethylene oxide makes possible an existence of ionic conductivity at anhydrous conditions [14] that widens the range of operating conditions and, accordingly, the sphere of their practical application. In [15] the use of a composite material based on polyethylene oxide (PEO) and lithium salts as a complex ion-conductive polymer system was proposed. The choice of PEO was caused by significant ability to solvate inorganic salts and, as a consequence, the presence of high ionic conductivity in materials on its basis [5]. As charge transport in polymer electrolytes passes mainly through the amorphous regions [4, 5, 16, 18], a high molecular weight of the crystalline PEO and complexes formation with alkali metal salts greatly reduce the ionic conductivity at room temperature [5] but provide opportunities of sufficiently high conductivity at elevated temperatures. In [22] a model of the mechanism of charge transfer that takes into account the accuracy of ion association in the PEO-salt (PEO-Li^+^) system was proposed. According to this model, the charge transport through the polymer matrix can occur in four ways:

1. The lithium cation motion along the single polymer chain of PEO
2. The lithium cation motion from one to another polymer chain
3. The lithium cation motion along the polymer chain of PEO between ion clusters (cation-anion)
4. Cation motion between ion clusters and the polymer chain

As noted in [5], the level of ionic conductivity is determined by three factors: the segmental mobility of the polymer chain, the concentration of charge carriers, and the number of motions for transmitting a cation (‘decoupling index’ [23]). For taking into account the effect of these factors on the overall electrical and dielectric properties of composite materials and finding an optimal balance between these factors, the chemical structure of a polymer should be taken into consideration. According to [16, 22] the presence of oxygen with a large electron-donor ability in PEO ether atoms promotes the formation of bonds with lithium cations and their movement through the polymer matrix in accordance to the mechanisms listed above. The last factor is very important in ensuring a high level of conductivity of the polymer system.

Besides, PEO epoxy oligomer of diglycide aliphatic ester of polyethylene glycol (DEG) also contains fragments with ether oxygen in the polymer chain. Its chemical structure is similar to the structure of PEO (Table 1) that makes possible to assume the transfer mechanism of lithium ions similar to PEO. However, DEG has good mechanical properties and heat resistance compared to PEO. Also, the amorphous structure of DEG allows the charge transport even at ambient conditions, while the high crystallinity of the PEO prevents achieving high level of ionic conductivity [5]. In [24] it is shown that such epoxy resins may be promising materials for ion-conductive materials creation.

**Table 1:** **Chemical structures of PEO and DEG**

Name	Chemical formula
PEO	
Epoxy oligomer of diglycide aliphatic ester of DEG	

Comparison of the chemical structures of PEO and epoxy oligomer of diglycide aliphatic ester of DEG.

Thus, the aim of the presented research was to study the electrical, dielectric, and thermal properties of epoxy polymer composites based on DEG with different contents of lithium salt LiClO_4_.

## Methods

The epoxy oligomer of diglycide aliphatic ester of DEG, Macromer, Vladimir, Russia and salt of lithium perchlorate (LiClO_4_, Sigma-Aldrich, USA) were used for synthesis of ion-conductive epoxy polymer composites. Since LiClO_4_ is hygroscopic and forms crystalline hydrates, it was previously pre-dried in vacuum at 80°C during 8 h. The chemical structure of aliphatic olіgomer DEG is shown in Table 1. After drying, the salt was dissolved in olіgomer DEG. Solutions of DEG-LiClO_4_ were prepared with LiClO_4_ content from 0 to 20 phr (parts per hundred) on 100 phr of DEG.

Polyethylene polyamine hardener (PEPA, Chimia, Kharkov, Ukraine) was used as a curing agent. DEG and PEPA contents were 90 and 10 phr, respectively, for all synthesized composites.

The thermal characteristics were studied by differential scanning calorimetry (DSC) at TA Instruments DSC Q2000 (TA Instruments, New Castle, DE, USA) in the temperature range from −70°C to +150°C with the heating rate of 10°C/min. Glass transition temperature (*T*_*g*_) was determined from the DSC curves at the second heating.

The electrical and dielectric characteristics of the synthesized composites were investigated by the broadband dielectric analyzer ‘Novocontrol Alpha’ with Novocontrol Quatro Cryosystem (Novocontrol Technologies, Montabaur, Germany) that was equipped with a two-electrode circuit, in the frequency range 10^−1^ to 10^7^ Hz and the temperature range from −60°C to +200°C. The voltage applied to a sample was equal to 0.5 V. The test samples had diameter of 20 mm and a thickness of 0.5 mm and were previously coated by aluminum under vacuum. The obtained data was analyzed using the software ‘Novocontrol WinDeta 3.8.’

The structural organization and features of macromolecular ordering of the synthesized polymer systems were investigated by wide-angle X-ray scattering (WAXS) using the X-ray diffractometer DRON-4.07 (Burevestnik, Saint-Petersburg, Russia). The X-ray optical scheme was performed using Debye-Scherrer method by passing the primary beam through the polymer sample polymer using Cu K_α_ emission (*λ* = 1,54 Å) that was made monochromatic using Ni filter. The X-ray tube BSV27Cu (*U* = 30 kV, *I* = 30 mA) was used as a source of characteristic X-ray irradiation. The investigations were carried out by automatic step scanning in the range of scattering angles (2*θ*) from 2.6° to 40°, and the exposure time was 5 s. The research temperature was *T* = 20°C ± 2°C.

All investigations were repeated three times for statistical data manipulation.

## Results and discussion

**Figure 1:**
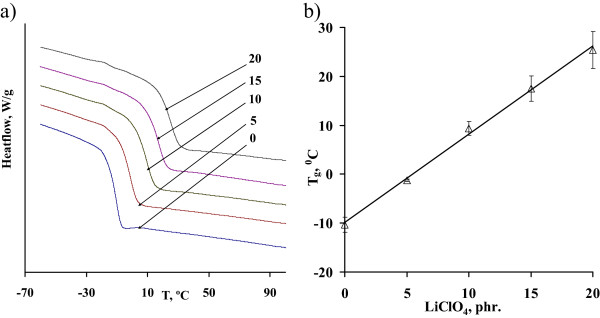
**DSC studies of the DEG/LiClO**_**4**_ **systems.** DSC curves **(a)** and dependence of the glass transition temperatures **(b)** of the DEG systems on LiClO_4_ content.

**Figure 2:**
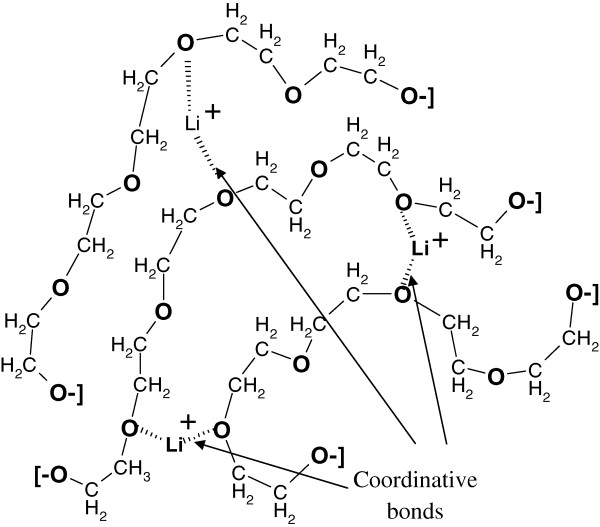
**Scheme of the DEG/LiClO**_**4**_ **system.** DEG/LiClO_4_ system consists of DEG polymer chains with coordinative bonds between lithium cations and ether oxygen.

The DSC results are shown in Figure 1a. The values of the glass transition temperature derived from the curves of the obtained composites depending on the content of LiClO_4_ in the reaction mixture are shown in Figure 1b. It is obvious that the increase of LiClO_4_ amount in reactive mixture from 0 to 20 phr leads to a linear increase of the glass transition temperature from −10°C to 25°C. This can be a result of the electrostatic interactions between lithium cations Li^+^ and the macromolecular r chain of DEG with immediate forming of coordinative complexes, such as {ether oxygen-lithium cations-ether oxygen} (Figure 2), which are accompanied by displacement of electron density of the oxygen atoms and their partial polarization [5, 21, 25]. The result is a substantial reduction of segmental mobility of DEG chains within the complexes formed that shows up in a glass transition temperature rise of polymer matrix. The linear dependence of *T*_*g*_ on the salt content testifies that all the lithium ions participate in the formation of coordination bonds.

The similar dependences were found in various sources. In [20] the growth of *T*_*g*_ from −46°C to −30°C at low salt content of LiClO_4_ in PEO (1 mol/kg) and the subsequent slow decrease to −35°C for the concentration of 7.5 mol/kg was reported. At the same time, according to [26], the addition of lithium salt LiPF_6_ in a PEO with high molecular weight reduces both glass transition temperature and melting point. The injection of another lithium salt LiCF_3_SO_3_ in PEO results in lowering of *T*_*g*_ from −65°C to −71°C [27]. In these cases, the coordination bonds were absent and, contrary to DEG, the molecular mobility of the polymer chains increases.

**Figure 3:**
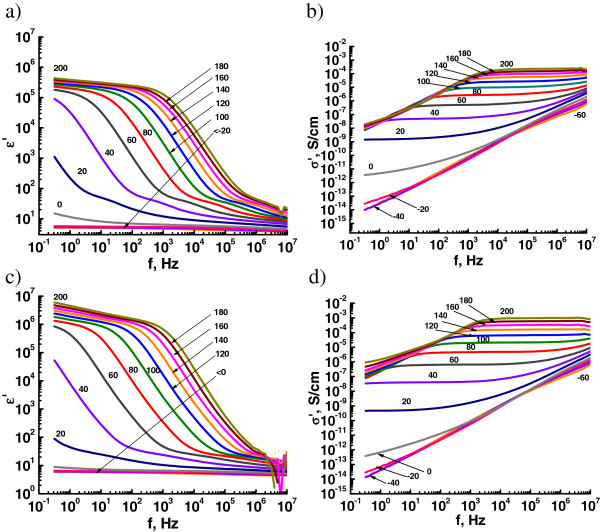
**BDS studies of the DEG/LiClO**_**4**_ **systems.** Permittivity **(а, c)** and real part of complex conductivity **(b, d)** spectra at different temperatures (from −60°C to +200°C) of the obtained composites with LiClO_4_ contents: **(a, b)** 5; **(c, d)** 10 phr.

**Figure 4:**
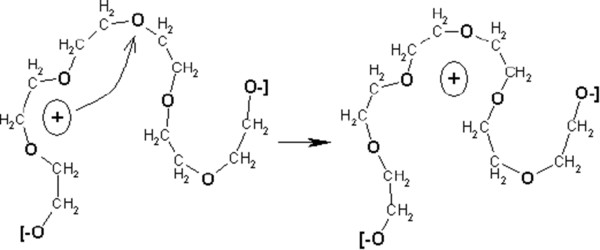
**Scheme of lithium cation transfer in the DEG/LiClO**_**4**_ **system.** The lithium cation transfer along the DEG polymer chain.

Figure 3 shows the isothermal spectra of the real part of the complex permittivity (*ϵ'*) and the conductivity (*σ'*) for different concentrations of LiClO_4_ in DEG obtained in the temperature range from −60°C to 200°C. One can see that values and character of the *σ'* and *ϵ'* curves depend on two factors: the content of LiClO_4_ and the temperature of measurements. At temperatures below the glass transition, the permittivity has low values and hardly varies with frequency indicating the ‘blocking effect’ of free charge carriers due to the low mobility of macromolecular chains of the polymer matrix. In the same temperature range, the values of the real part of the complex conductivity vary linearly with frequency, i.e., such systems are insulators. At temperatures higher than *T*_*g*_, ‘defrosting’ of the polymer chains occurs that leads to the release of lithium cations and growth of *ϵ'* values. Free lithium cations pass into the conducting band and begin to move along the polymer chain through the interactions with oxygen ether atoms, which exist in macromolecular chains (Figure 4). This charge transfer leads to an increase in the electrical conductivity of the systems and the appearance of a plateau at low frequencies (the so-called DC conductivity plateau, an isotherms area, where conductivity values are independent on frequency) on the spectra of the real part of the complex conductivity. The *σ′* dependence on angular frequency *ω =2*π*f* is described by the following equation [28, 29]:

1

where *σ*_0_ is the conductivity that is independent on frequency, the exponent factor *s* equals 0 < *s* ≤ 1, and *А* is a numeric factor. The contribution of the second part is insignificant at low frequencies, and the conductivity, which is independent on frequency (plateau on the graph), is associated with the DC conductivity. At high frequencies above the critical frequency *f*_*c*_, the main role is played by the second frequency-dependent parameter *σ*_*ac*_(*ω*) *~ ω*^*s*^ that characterizes the jumping conductivity in a disordered solid state (AC conductivity). The critical frequency *f*_*c*_ and the parameters *A* and *s* in the Equation 1 depend on the temperature and the conductivity of the systems [29].

The values of the permittivity *ϵ′* at frequency 10^3^ Hz are represented in Table 2. The values of the real part of the complex conductivity *σ′* for different temperatures throughout the range of the lithium salt content in the epoxy resin are also presented.

**Table 2:** **Characteristics of epoxy polymers with different content of LiClO**_**4**_

Content LiClO _4_ , phr	ϵ′ (10 ^3^ Hz) ^a^			
	σ′ (S/cm)/ ***σ***_***dc***_ (S/cm) ^b^			
	60°С	100°С	160°С	200°С
0	36 1.7 × 10^−7^/1.52 × 10^−7^	254 1.4 × 10^−6^/1.47 × 10^−6^	3,220 8.1 × 10^−6^/7.9 × 10^−6^	6,040 1.3 × 10^−5^/1.21 × 10^−5^
5	68 4.7 × 10^−7^/5.1 × 10^−7^	3,140 1.0 × 10^−5^/1.01 × 10^−5^	60,800 9.6 × 10^−5^/9.85 × 10^−5^	128,000 2.3 × 10^−4^/2.3 × 10^−4^
10	48 6.96 × 10^−7^/6.5 × 10^−7^	2,720 1.97 × 10^−5^/2.08 × 10^−5^	208,000 3.2 × 10^−4^/3.16 × 10^−4^	680,000 9.97 × 10^−4^/1.01 × 10^−3^
20	33 1.5 × 10^−7^/1.48 × 10^−7^	1,650 1.2 × 10^−5^/1.17 × 10^−5^	212,000 3.2 × 10^−4^/3.26 × 10^−4^	626,000 1.1 × 10^−3^/1.17 × 10^−3^

^a^Experimental error on ϵ′ was ±1; ^b^experimental error on σ′ was ±1 × 10^−10^ S/cm. Electrical and dielectric characteristics of the epoxy systems with different contents of LiClO_4_ at different temperatures.

A number of free charge carriers, namely, the lithium ions Li^+^, which overcome the energy barrier and move into conductive state, grow with further increase of the temperature (above 40°C to 60°C). This leads to the blocking effect of electrodes that is caused by the space charge polarization. The blocking effect is manifested in appearance of a plateau on the *ϵ'* isotherms at low frequencies and falling values of the real part of the complex conductivity left from plateau of the DC conductivity. The dominance of the conductivity relaxation is observed at high frequencies [17].

**Figure 5:**
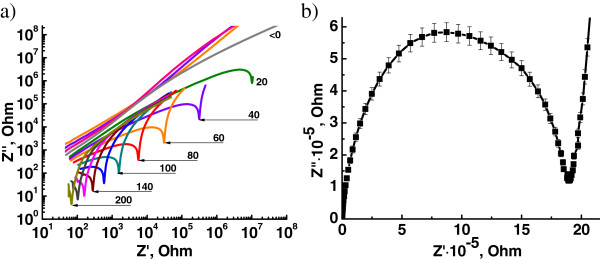
***Z*** **″/** ***Z*** **′ plots for the DEG/LiClO**_**4**_ **systems.** *Z*″/*Z*′ plots for epoxy system with 5 phr of LiClO_4_ in the temperature range from −60°C to +200°C in double logarithmic coordinates **(a)** and in Cole-Cole coordinates at 30°C **(b)**.

The frequency dependences of the impedance of the systems studied on temperature were also analyzed. Figure 5a shows the isothermal spectra of *Z" = f*(*Z′*), where *Z′ = M″*/(*ω · C*_0_) is the real part of the complex impedance, *Z″ = M′*/(*ω · C*_0_) is the imaginary part of the complex impedance, *М*′ and *М″* are the real and the imaginary parts of electrical modulus, *C*_0_ is the cell capacitance without the sample in vacuum, in double logarithmic coordinates for the DEG system containing 5 phr LiClO_4_ in the temperature range from −60°C to +200°C. It is evident that the nature of the isotherms at temperatures below the glass transition temperature *T*_*g*_ corresponds to the character of the open Warburg diffusion impedance (direct linear relationship) that describes a semi-infinite diffusion process and due to the ‘frosting process’ of charge transfer in such systems. The bulk resistance of the systems is represented by minimums on isotherms, which appears when the temperature passes the glass transition temperature of the systems. The curves acquire the form of a finite (closed) Warburg diffusion impedance that describes the linear diffusion process in a homogeneous layer of finite thickness, i.e., the charge transfer through the bulk of the systems becomes ‘unfrozen’ [30].

Cole-Cole plots (*Z″ = f*(*Z*′)) were built for calculating the DC conductivity *σ*_*dc*_. Figure 5 shows the classical Cole-Cole plots built for the composite of DEG with 5 phr LiClO_4_ at temperature 30°C. The dependence *Z″ ~ Z*′ forms a clear minimum at a certain value of *Z*′ in Cole-Cole coordinates. Conductivity values were calculated from the Equation 2:
2

where *R*_*dc*_ is a bulk resistance of the system (Ohm) that equals the value of *Z′* on the minimum of the Cole-Cole plot; *l* is a thickness of the sample (cm), and *S* is an area of the sample (cm^2^). The right part of the curve corresponds to surface polarization effects, which are observed in the low-frequency region. The left part of the curve corresponds to volume polarization effects in the high-frequency region. The calculated values of the conductivity *σ*_*dc*_ are presented in Table 2. The identity of the values of the conductivity *σ*_*dc*_ calculated with impedance analysis and the conductivity *σ′* values defined by plateau on the primary experimental spectra of the real part of the complex conductivity prove the eligibility of such analysis approaches for studying the behavior of the ion-conductive systems in wide temperature and frequency ranges, and validity of the obtained values of the conductivity.

These data suggest that ion-conductive systems based on DEG and LiClO_4_ are of interest as a solid polymer electrolyte able to operate at high temperatures (up to 200°C). According the TGA results, the weight loss at this temperature is negligible; thus, their usability at high temperatures is possible. At the same time, authors of [26] showed that a quite significant weight loss occurs for the polymer electrolyte based on PEO with the lithium salt LiPF_6_ (15% for the composite PEO-20% LiPF_6_).

On the other hand, a high level of the conductivity (approximately 1 × 10^−3^ S/cm) obtained at 200°C decreases rapidly with cooling so the conductivity at 100°C is two orders of magnitude lower than at 200°C. Apparently, the reason for that is the presence of coordinative bonds between lithium ions and DEG macromolecules, which are more stable at low temperatures and reduce the molecular mobility of the polymer chains. This can lead to a drop of ‘decoupling index’ (index of lithium ion mobility by jumping from one to another oxygen atom). Also, the formation of ion pairs [5] in the systems with increased contents of LiClO_4_ in the epoxy resin (higher than 20 phr) reduces the number of free charge carriers in the systems and, consequently, decreases the electrical conductivity and the permittivity. So, the conductivity of the system DEG with 20 phr LiClO_4_ at 60°C is on the same level as for pure DEG. However, this effect is significant only at temperatures up to 100°C. A rapid growth of the electrical conductivity and the permittivity due to the destruction of aggregates that is accompanied by release of charge carriers, namely lithium cations Li^+^, occurs when the temperature rises. The maximum values of the conductivity *σ*′ = 1.1 × 10^−3^ S/cm and the permittivity *ϵ'* = 6.3 × 10^5^ are attained for the system DEG with 20 phr of LiClO_4_ at 200°C (Table 2).

The possible way to improve the conductivity at low temperatures is the injection of carbon nanotubes, which form a kind of ‘trunks’ that facilitate the transport of ions, in a polymer matrix [18, 26]. In [26] the same level of the conductivity (approximately 10^−3^ S/cm) in the presence of 5% carbon nanotubes has been reached at 50°C to 100°C. Other types of nanofillers also give a positive effect [5, 21]. In some cases, various plasticizers are injected in PEO to improve the transport of lithium ions [26, 27].

**Figure 6:**
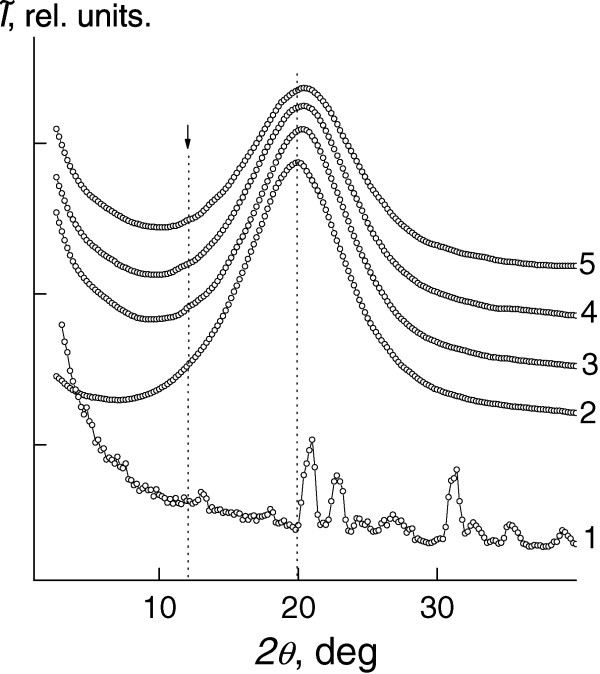
**The WAXS studies of the DEG/LiClO**_**4**_ **systems.** The wide-angle X-ray diffraction patterns of a salt of LiClO_4_ (1) and DEG/LiClO_4_ systems with the salt content: 0 phr (2), 5 phr (3), 10 phr (4), and 20 phr (5) at 20°C ± 2°C.

The analysis of the wide-angle X-ray diffraction patterns of the systems showed that all of them are amorphous (Figure 6). In particular, epoxy oligomer DEG that was cured with polyethylene polyamine is characterized by short-range ordering in the space translation of molecular fragments of its cross-site links. That is confirmed by presence of one diffraction peak (calculated from the angular half-width) of the diffusion type (amorphous halo), in which the angular position (2*θ*_*m*_) is about 20.0°.

The average value of the period (*d*) of a short-range molecular ordering of DEG internodal molecular segments in a polymer volume can be calculated using Bragg equation:
3

where *λ* is the wavelength of the characteristic X-ray emission (*λ* = 1.54 Å for Сu K_α_ emission) and it equals to 4.44 Å.

However, the introduction of LiClO_4_ salt that has a crystalline structure into the epoxy resin is accompanied by changes in the diffraction pattern. This is evidenced by the presence of subtle diffraction peak of the diffuse type at 2*θ*_*m*_ ≈ 12.2° on the background of the amorphous halo, which is similar to the angular position of the DEG at 2*θ*_*m*_ ≈ 20.0° (*d* ≈ 4.44 Å). According to [31], this diffraction peak characterizes the existence of metal-polymer complexes of the donor-acceptor type, in our case, between central ions (Li^+^) and ether oxygen of the epoxy chains in the intermolecular volume of the epoxy resin.

The gradually increasing LiClO_4_ content from 0 to 20 phr in the volume of epoxy resin leads to the displacement of the amorphous halo at 2*θ*_*m*_ ≈ 20.0°, which characterizes the short-range order of fragments of the DEG internodal molecular segments, in the region of large scattering angles. That indicates a tendency to decrease the Bragg distance between the molecular segments (Table 3).

**Table 3:** **The Bragg distance between the molecular segments of epoxy polymers with different contents of LiClO**_**4**_

Content LiClO _4_ , phr	2 ***θ***_***m***_ ***,*** degrees ^a^	***d*** , Å
Pure LiClO_4_ (*for comparison*)	20.0	4.44
0	20.0	4.44
5	20.2	4.39
10	20.4	4.35
20	20.4	4.35

^a^Experimental error on 2*θ*_*m*_ was ±0.05°.

## Conclusions

Thus, the synthesis of epoxy polymers in the presence of LiClO_4_ was made possible to obtain an ion-conductive polymeric material with a high level of ionic conductivity (approximately 10^−3^ S/cm) and the permittivity (6 × 10^5^) at elevated temperatures (200°C). The presence of ether oxygen atoms in polymer chains of the aliphatic epoxy DEG makes its structure similar to the PEO structure and provides the possibility for lithium cations transfer throughout the ether oxygen atoms. Contrariwise, epoxy polymer DEG has a higher heat resistance comparing to PEO, thus it is of interest as a solid polymer electrolyte able to operate at high temperatures.

## Authors’ information

ML is a PhD student at the Institute of Macromolecular Chemistry of the National Academy of Sciences of Ukraine (NAS of Ukraine). MI is a Doctor in Physics, senior staff scientist at the Institute of Macromolecular Chemistry of the NAS of Ukraine. YeM is a Professor, Dr. Hab. in Polymer Physics and PhD in Macromolecular Chemistry, leading staff scientist at the Institute of Macromolecular Chemistry of the NAS of Ukraine, Director of the Centre for Thermophysical Investigations and Analysis of the NAS of Ukraine. MO is a junior researcher at the Institute of Macromolecular Chemistry of the NAS of Ukraine. DV is a PhD in Polymer Physics, researcher at the Institute of Macromolecular Chemistry of the NAS of Ukraine. EL is Professor, Dr. Hab in Macromolecular Chemistry, Head of Department at the Institute of Macromolecular Chemistry of the NAS of Ukraine. GB is a Dr. Hab. in Physics, Director of Research CNRS, Université de Lyon, Université Lyon 1, Ingénierie des Matériaux Polymères, UMR CNRS 5223, IMP@LYON1. AS is a Doctor in Physics, researcher DR2 at Université de Lyon, Université Lyon 1, Ingénierie des Matériaux Polymères, UMR CNRS 5223, IMP@LYON1.

## Abbreviations

AC: alternating current
BDS: broadband dielectric spectroscopy
DC: direct current
DEG: epoxy oligomer of diglycide aliphatic ester of polyethylene glycol
DSC: differential scanning calorimetry
LiClO_4_: salt of lithium perchlorate
PEO: polyethylene oxide
PEPA: polyethylene polyamine
PWA: phosphotungstic acid
*T*_*g*_: glass transition temperature
TGA: thermogravimetric analysis
WAXS: wide-angle X-ray scattering.

## References

[1] Mamunya Y, Iurzhenko M, Lebedev E, Levchenko V, Chervakov O, Matkovska O, Sverdlikovska O (2013). Electroactive polymer materials.

[2] Qi L, Dong SJ (2007). Organic/inorganic nanocomposite polymer electrolyte. Chin Chem Lett.

[3] Pradhan DK, Choudhary RNP, Samantaray BK (2008). Studies of structural, thermal and electrical behavior of polymer nanocomposite electrolytes. Express Polym Lett.

[4] Zhukovsky VM, Bushkov OV, Lyrova BI, Tyutyunnik AP, Anymytsa IE (2001). The problem of fast ion transport into solid polymer electrolytes. Rus Chem J.

[5] Lysenkov EA, Klepko VV (2011). Nanocomposite polymer electrolytes: structure and properties. Ukr Polym J.

[6] Thakur AK, Pradhan DK, Samantaray BK, Choudhary RNP (2006). Studies on an ionically conducting polymer nanocomposite. J Power Sources.

[7] Garnier FH (1989). Conducting polymers. Prog Phys Sci.

[8] Trachtenberg LI, Gerasimov GN, Potapov VK, Rostovschykova TN, Smirnov VV, Zufman VY (2001). Sensor, catalytic and electrical properties of the nanocomposite metal polymeric films. Mosc Univ Vestnik Seria2 (Chemistry).

[9] Chen Y, Kim H (2009). Silyl functionalized poly(vinylidene fluoride)/phosphotungstic acid/aminopropyltrimethoxysilane ternary composite membrane for proton conductivity. J Power Sourc.

[10] Zhang H, Zhu B, Xu Y (2006). Composite membranes of sulfonated poly(phthalazinone ether ketone) doped with 12-phosphotungstic acid (H_3_PW_12_O_40_) for proton exchange membranes. Sol State Ion.

[11] Colicchio I, Wen F, Keul H, Simon U, Moeller M (2009). Sulfonated poly(ether ether ketone)–silica membranes doped with phosphotungstic acid. Morphology and proton conductivity. J Membr Sci.

[12] Cui Z, Liu C, Lu T, Xing W (2007). Polyelectrolyte complexes of chitosan and phosphotungstic acid as proton-conducting membranes for direct methanol fuel cells. J Power Sourc.

[13] He R, Li Q, Xiao G, Bjerrum NJ (2003). Proton conductivity of phosphoric acid doped polybenzimidazole and its composites with inorganic proton conductors. J Membr Sci.

[14] Shevchenko V, Klimenko N, Stryutskyy O, Lisenkov E, Vortman M, Rudakov V (2011). Synthesis and properties of organic–inorganic polymer proton-conducting membranes based on amine oligoether precursors. Ukr Chem J.

[15] Armand AM, Chabagno JM, Duclot M (1978). Second International Meeting on Solid Electrolytes: 1978 September 20–22 Book Extended Abstract.

[16] Klepko V, Zhyhir O (2008). Relaxation properties of polymer electrolytes based on block-copolymers PEO-PPO-PEO. Polym J.

[17] Fomenko AA, Gumenna МА, Klimenko NS, Shevchenko V, Klepko VV (2008). Dielectric properties and conductivity of hybrid organic–inorganic systems based on polypropyleneglycol and POSS. Ukr Polym J.

[18] Lisenkov EА, Gomza YP, Davydenko VV, Klepko VV, Rehteta MA, Kunitsky UA, Shabelnik IM (2010). Effect of anisometric nanofillers on structure and properties of polymer electrolytes based on polypropylene. Nanosyst Nanomater Nanotechnol.

[19] Stepanenko OM, Reiter LG, Ledovskih VM, Ivanov SV (2000). General and Inorganic Chemistry.

[20] Wieczorek W, Raducha D, Zalewska A, Stevens JR (1998). Effect of salt concentration on the conductivity of PEO-based composite polymeric electrolytes. J Phys Chem B.

[21] Lisenkov EА, Gomza YP, Klepko VV (2010). Effect of anisometric nanofillers on structure and conductivity of PEG1000/LiClO_4_ in bulk and thin films. Ukr Polym J.

[22] Gray FM (1997). Polymer Electrolytes.

[23] Torell LM, Angell CA (1988). New conducting polymer. Br Polym J.

[24] Matkovska O, Mamunya Y, Shandruk M, Zinchenko O, Lebedev E, Boiteux G, Serghei A (2013). Proton-conductive epoxy polymers: influence of structure on electrical properties. Ukr Polym J.

[25] Kiselev YM (2008). Coordination Chemistry.

[26] Ibrahim S, Johan MR (2012). Thermolysis and conductivity studies of poly(ethylene oxide) (PEO) based polymer electrolytes doped with carbon nanotube. Int J Electrochem Sci.

[27] Johan MR, Ting LM (2011). Structural, thermal and electrical properties of nano manganese-composite polymer electrolytes. Int J Electrochem Sci.

[28] Kremer F, Schonhals A (2003). (Eds): Broadband Dielectric Spectroscopy.

[29] Psarras GC, Manolakaki E, Tsangaris GM (2003). Dielectric dispersion and ac conductivity in iron particles loaded polymer composites. Compos Part A.

[30] Pershina KD, Kazdobin KO (2012). The Impedance Spectroscopy of the Electrolytic Materials.

[31] Shtompel VI, Kercha YY (2008). Structure of Linear Polyurethanes.

